# Risk Factors and Outcomes of Hemorrhagic Transformation in Acute Ischemic Stroke Following Thrombolysis: Analysis of a Single-Center Experience and Review of the Literature

**DOI:** 10.3390/medicina61040722

**Published:** 2025-04-14

**Authors:** Ileana Neacă, Cristina Elena Negroiu, Iulia Tudorașcu, Raluca Dănoiu, Cristiana Gianina Moise, Despina Manuela Toader, Suzana Dănoiu

**Affiliations:** 1 Doctoral School, University of Medicine and Pharmacy of Craiova, 200349 Craiova, Romania; 2 Sanocare Medical Center Craiova, 200061 Craiova, Romania; danoiuraluca@yahoo.ro; 3 Department of Pathophysiology, University of Medicine and Pharmacy of Craiova, 200349 Craiova, Romania; cristina.negroiu@yahoo.ro (C.E.N.); suzanadanoiu@yahoo.com (S.D.); 4 Department of Physiology, University of Medicine and Pharmacy of Craiova, 200349 Craiova, Romania; cristiana.moise10@gmail.com; 5 County Clinical Emergency Hospital of Craiova, 200642 Craiova, Romania; despinamtoader@yahoo.com

**Keywords:** acute ischemic stroke, intravenous thrombolysis, hemorrhagic transformation, risk factors

## Abstract

*Background and Objectives*: This is a retrospective study conducted at the Clinical County Hospital of Craiova, Romania, providing valuable insights into hemorrhagic transformation (HT) in thrombolyzed patients with acute ischemic stroke (AIS). Hemorrhagic complications remain a significant concern after intravenous thrombolysis with recombinant tissue plasminogen activator (rt-PA). This study aims to analyze clinical and biological factors associated with HT following thrombolysis. *Materials and Methods*: A retrospective analysis was conducted on 356 patients who received rt-PA at the Clinical County Hospital of Craiova between January 2020 and December 2024. Patients were divided into three groups based on CT findings at 24 h post-thrombolysis: no HT, minimal HT, and massive HT. Baseline characteristics were analyzed, including demographics, medical history, NIHSS scores, imaging findings, and laboratory parameters. Statistical analysis was performed using ANOVA and chi-square tests, with a significance threshold of *p* < 0.05. *Results*: HT occurred in 12.08% of patients (minimal HT: 8.15%, massive HT: 3.93%). Mortality was significantly higher in the massive HT group (71.43%) compared to minimal HT (41.38%) and non-HT (13.42%) (*p* < 0.001). Lower platelet count (*p* = 0.003), elevated blood glucose (*p* = 0.004), prolonged QT interval (*p* = 0.004), and reduced fibrinogen levels (*p* = 0.005) were significantly associated with HT. Other risk factors included atrial fibrillation (*p* = 0.001), hypertension (*p* = 0.005), delayed door-to-needle time (*p* < 0.001), diabetes mellitus (*p* = 0.007), dense ACM sign on CT (*p* = 0.003), older age (*p* < 0.001), obesity (*p* = 0.001), early neurological deterioration at 2 h/24 h (*p* < 0.001), elevated GOT (*p* < 0.001), elevated GPT (*p* = 0.002), lower LDL cholesterol (*p* < 0.001), lower total cholesterol (*p* = 0.001), and lower triglycerides (*p* < 0.001). *Conclusions*: Patients with HT had worse clinical outcomes, with massive HT associated with the highest mortality. Risk factors include age, nutritional status, hyperglycemia, and low platelet and fibrinogen levels, among others.

## 1. Introduction

The incidence rates of acute ischemic stroke (AIS) are similar to those of acute coronary syndromes [1]. Both of these acute ischemic syndromes are responsible for the vast majority of deaths from cardiovascular diseases and, consequently, for total mortality in most countries [1]. AIS is a medical emergency that occurs when reduced blood flow to the brain leads to brain cell damage [2]. The primary cause is the obstruction of a cerebral artery, most commonly due to a thrombus forming on an atherosclerotic plaque or an embolus originating from the heart, often in the context of atrial fibrillation or other cardioembolic conditions [3]. Other causes are represented by small vessel arteriolosclerosis, and for younger patients, the causes are even more diverse, reaching from extracranial dissection to Moyamoya disease, fibromuscular dysplasia, or different hypercoagulable states [4,5].

Thrombolytic therapy seeks to break down the thrombus and restore local blood circulation. Recombinant tissue plasminogen activator (rtPA) is administered by infusion at a dose of 0.9 mg/kg (maximum dose of 90 mg) over 60 min, with 10% of the dose given as a bolus over the first minute [6]. Recent Canadian stroke treatment guidelines allow the use of tenecteplase as an alternative to alteplase within 4.5 h of last known well (LKW), as studies have shown it is not inferior to alteplase [7]. To minimize complications, selecting the right patients is crucial. Just as important is the immediate monitoring period following lysis. During this time, the patient should be admitted to an intensive care or stroke unit, undergo frequent clinical assessments, and receive prompt treatment for any emerging symptoms, especially hypertension [7].

Hemorrhagic transformation (HT) is a frequent complication in patients with AIS, characterized by the leakage of peripheral blood into the brain due to the disruption of the blood–brain barrier (BBB) following the ischemic event [8]. It is often aggravated by reperfusion treatments such as recombinant tissue plasminogen activator or endovascular therapy [9].

Extensive parenchymal hematomas, present in approximately 6% of patients following intravenous thrombolysis, are among the most critical complications due to their high mortality rate [10]. The reported incidence of HT varies between studies. For instance, a study on 214 patients with ischemic stroke found an HT rate of 14.0% in those treated with t-PA [11], while another study involving 285 stroke patients reported an incidence of 25.3% following thrombolytic therapy [12].

The development of HT is a complex process influenced by various inflammatory factors and signaling pathways [13]. The disruption of the BBB is regarded as the key factor contributing to the development of HT [14]. During AIS, microglia become activated and differentiate into M1-type cells, releasing inflammatory factors and MMP-9, which enhance BBB permeability. This is followed by the migration and infiltration of peripheral inflammatory cells into the brain, where they generate free radicals, MMP-9, and other inflammatory mediators [13]. These interconnected inflammatory processes contribute to BBB disruption, ultimately increasing the risk of HT.

The occurrence of HT is influenced by a variety of factors, including epidemiological aspects (such as age, pre-stroke treatments, and underlying conditions), infarct characteristics (such as ischemic core size and timing of follow-up), acute-phase reperfusion methods (including intravenous thrombolysis and mechanical thrombectomy), radiological diagnostic tools (such as computed tomography and magnetic resonance imaging), and the use of antithrombotics in the post-acute phase [10].

This retrospective study aimed to identify and analyze clinical and biological factors associated with HT in thrombolyzed patients with AIS in Oltenia, Romania. Given that HT is a common and serious complication following intravenous thrombolysis with a recombinant tissue plasminogen activator, this study sought to evaluate potential risk factors—such as metabolic factors, comorbidities, and thrombolysis timing—that may contribute to its occurrence and severity. By recognizing these factors, this study aimed to improve early risk stratification, optimize treatment strategies, and ultimately enhance patient outcomes.

## 2. Materials and Methods

This study was approved by the Ethics Committee of the County Emergency Clinical Hospital Craiova (approval number 42473/20 September 2024), and informed consent was obtained from all patients or, when this was not possible, from their legal representatives.

A total of 356 patients who received thrombolysis at the Clinical County Hospital of Craiova was analyzed, considering the indications for thrombolysis, presentation within the optimal window of up to 4.5 h, and the existing contraindications. These patients were investigated from the beginning of 2020 until the end of 2024. No patients underwent endovascular thrombectomy, as our center does not have yet the capability for interventional treatment. Therefore, the analysis was conducted exclusively on cases treated with intravenous rt-PA.

These patients were divided into three groups based on the results of the CT evaluation at 24 h: a group of patients without HT, a group with minimal HT, and a group with massive HT. According to the established protocol, the classification includes four subtypes: hemorrhagic infarction type 1 (IH1), hemorrhagic infarction type 2 (IH2), parenchymal hematoma type 1 (HP1), and parenchymal hematoma type 2 (HP2). Patients with IH1, IH2, and HP1 (characterized by no mass effect or minimal mass effect) were categorized as having minimal HT, while those with HP2 (associated with mass effect) were classified as having massive HT. This classification provides a clinically and radiologically relevant simplification, facilitating interpretation and application in medical practice.

rt-PA was administered via intravenous infusion at a dose of 0.9 mg/kg body weight, with a maximum dose of 90 mg.

Baseline patient data were analyzed including demographics; family and personal medical history; NIHSS score at admission, 2 h, 24 h, 7 days, and discharge; time from symptom onset to rt-PA administration; CT scan findings at admission and 24 h; ECG findings; and biological parameters, as well as medication at admission and discharge. Regarding the analyzed biological data, the values for platelet count, INR, and blood glucose were collected in the emergency department, immediately upon the patient’s arrival, as they directly influenced the decision to initiate thrombolytic therapy. The remaining biological parameters were obtained within the first 24 h of admission to the neurology department and processed by the hospital laboratory.

The inclusion criteria were age over 18 years, clinical diagnosis of ischemic stroke, and symptom onset within 4.5 h of presentation. The exclusion criteria included cerebral hemorrhage on CT; symptom onset beyond 4.5 h or unknown onset; mild or rapidly resolving neurological symptoms; history of stroke or head trauma in the last 3 months; symptoms suggestive of subarachnoid hemorrhage; history of aneurysm, arteriovenous malformations, or intracranial neoplasms; recent brain or spinal surgery (within the last 14 days); major surgery (abdominal, thoracic, vascular) within the last 14 days; severe treatment-resistant hypertension (SBP > 185 mmHg or DBP > 110 mmHg); hypoglycemia (glucose < 50 mg/dL); active internal bleeding or known coagulation disorders; INR > 1.7; thrombocytopenia (platelets < 100,000/mm^3^); recent gastrointestinal bleeding (within the last 21 days); arterial puncture in a non-compressible site within the last 7 days; recent non-vitamin K oral anticoagulant (NOAC) therapy within the last 48 h; triple antithrombotic therapy; low-molecular-weight heparin in therapeutic doses within the last 24 h; recent myocardial infarction (≥6 h–7 days); suspected or known aortic dissection; suspected or confirmed infective endocarditis; and recent childbirth (natural delivery within the last 10 days or cesarean section within the last 14 days).

### Statistical Analysis

Quantitative data with normal distribution are presented as mean ± standard deviation, and differences between groups were compared using the ANOVA test. Categorical variables are expressed as proportions and were compared using the chi-square test. A *p* value of <0.05 was considered statistically significant. All statistical analyses were performed using Excel 2016.

## 3. Results

### 3.1. Hemorrhagic Transformation and Patient Outcomes

**Figure 1:**
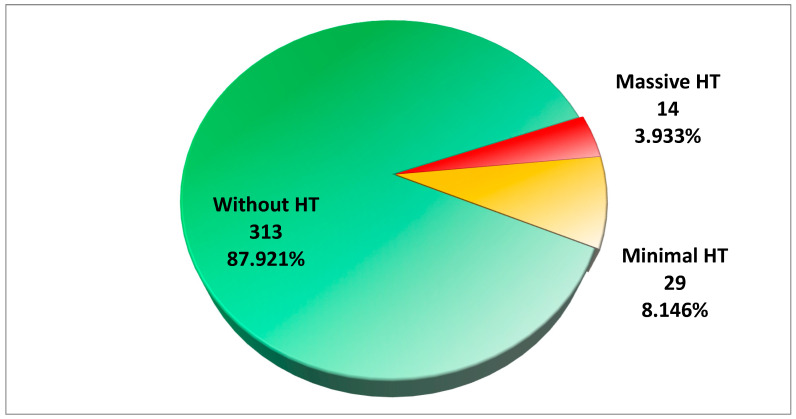
The hemorrhagic transformation of thrombolyzed ischemic stroke.

Most of the 356 patients analyzed (87.92%) did not experience HT. A smaller proportion had minimal HT (8.15%) or massive HT (3.93%). The mortality rate was significantly higher in patients with HT, especially those with massive HT (*p*-value of <0.001) (see Table 1 and Figure 1).

### 3.2. Age Distribution and Comorbidities

**Figure 2:**
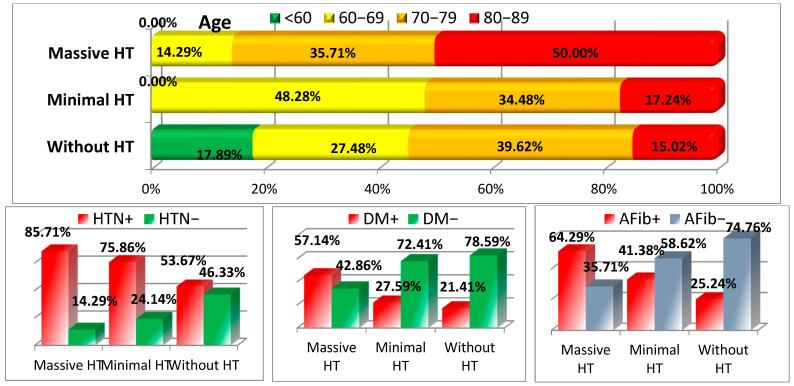
Age distribution and comorbidities (HTN—hypertension, DM—diabetes mellitus, AFib—atrial fibrillation).

The age distribution showed a clear trend: younger patients (<60 years) were predominantly found in the group without HT (17.89%), while minimal and massive HT were more common among older patients, particularly those aged 80–89 years (17.24% in minimal HT and 50% in massive HT). A significant difference was observed between the age groups with a *p*-value of <0.001. Furthermore, comorbidities such as hypertension, diabetes mellitus, and atrial fibrillation were more prevalent in the HT groups. Hypertension was present in 75.86% and 85.71% of patients with minimal and massive HT, respectively, compared to 53.67% in the group without HT (*p* = 0.005). Similarly, diabetes mellitus and atrial fibrillation showed higher frequencies in the HT groups, particularly massive HT (see Figure 2 and Table 1).

### 3.3. Nutritional Status and Risk Factors

**Figure 3:**
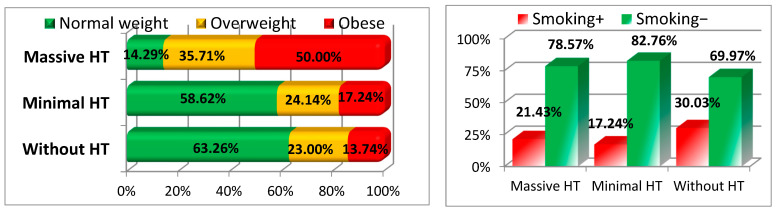
Nutritional status and smoking.

Nutritional status appeared to influence the likelihood of HT. The majority of patients in the group without HT were of normal nutritional status (63.26%), while only 14.29% of patients with massive HT had normal nutritional status. On the other hand, a higher percentage of patients with massive HT were obese (50%), compared to only 13.74% in the group without HT. Although smoking status did not show a significant difference (*p* = 0.287), it is noteworthy that patients with massive HT had a slightly lower smoking prevalence (21.43%) compared to those without HT (30.03%) (see Figure 3 and Table 1).

### 3.4. Imaging Findings

**Figure 4:**
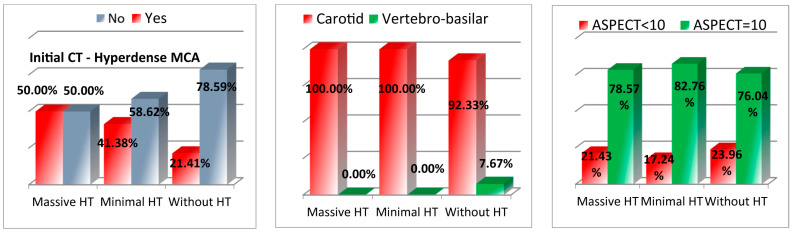
Imaging findings. (MCA = middle cerebral artery, ASPECT = Alberta stroke program early CT score).

Imaging data also revealed distinct patterns among the groups. Dense CT findings in the anterior circulation were more prevalent in the minimal (41.37%) and massive HT groups (50%) compared to the non-HT group (21.4%), with a significant *p*-value of 0.003. In contrast, no vertebro-basilar involvement was seen in patients with minimal or massive HT. ASPECT scores, which are used to assess early infarct volumes, were similar across the groups, with the majority of patients having an ASPECT score of 10 (see Figure 4 and Table 1).

### 3.5. Timing of Symptom Onset and Treatment

**Figure 5:**
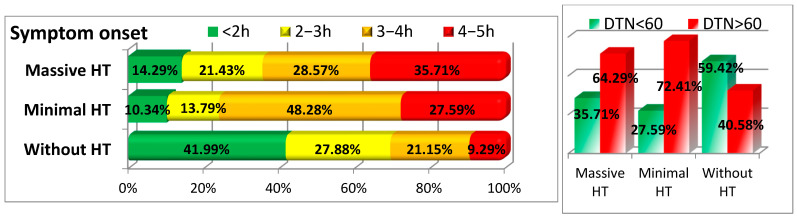
Timing of symptom onset and treatment ((DTN = door-to-needle time).

The timing of symptom onset relative to treatment initiation was a crucial factor. Patients with HT, especially those with massive HT, experienced longer delays in symptom presentation. Only 10.34% of the minimal HT group and 14.29% of the massive HT group presented within 2 h of symptom onset, compared to 41.99% in the group without HT (*p* < 0.001). Moreover, a significant delay was observed in the time to thrombolysis (DTN), with only 27.59% and 35.71% of patients with minimal and massive HT receiving thrombolysis within 60 min, as compared to 59.42% in the group without HT (*p* < 0.001) (see Figure 5 and Table 1).

### 3.6. Laboratory and Clinical Parameters by Type of Hemorrhagic Transformation

#### 3.6.1. QT Interval

**Figure 6:**
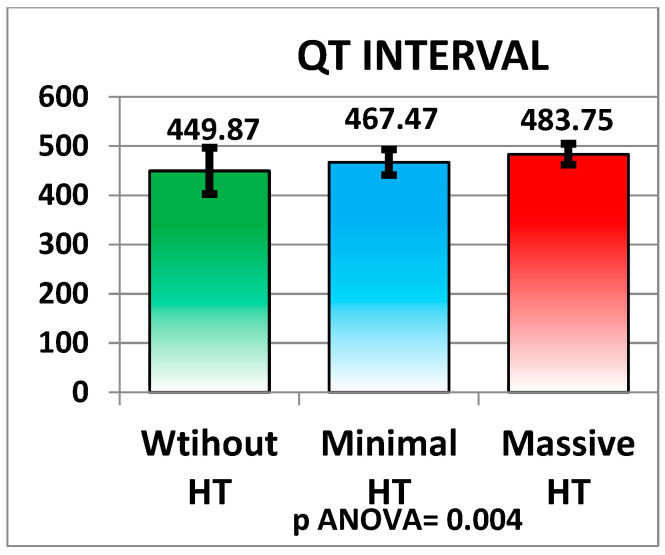
Interval QT.

Significant differences in laboratory and clinical parameters were observed among patients with different types of HT. One notable finding was the QT interval, which was significantly longer in patients with massive HT (483.75 ± 21.24 ms) compared to those without HT (449.87 ± 47.04 ms), with a *p*-value of 0.004. The QT interval was also prolonged in the minimal HT group (467.47 ± 25.85 ms) when compared to the non-HT group. These findings suggest a potential association between a prolonged QT interval and more severe forms of HT (see Figure 6 and Table 2).

#### 3.6.2. Metabolic and Hematological Changes

**Figure 7:**
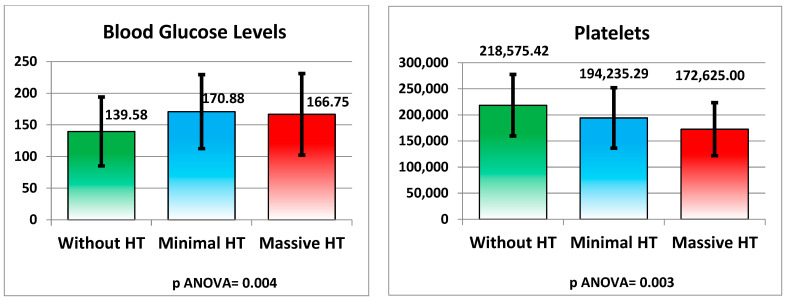
Metabolic and hematological changes.

Several metabolic and hematological parameters were significantly different across the groups. Blood glucose levels were higher in patients with minimal HT (170.88 ± 58.43 mg/dL) and massive HT (166.75 ± 64.43 mg/dL) compared to those without HT (139.58 ± 54.46 mg/dL), with a *p*-value of 0.004. Platelet count was also significantly lower in the groups with HT, particularly in the massive HT group (172,625.00 ± 50,793.52 mg/μL), compared to the group without HT (218,575.42 ± 58,885.04 mg/μL), with a *p*-value of 0.003 (see Figure 7 and Table 2).

#### 3.6.3. Liver Function and Lipid Profile

Liver enzymes differed significantly between groups, with elevated GOT (AST) and GPT (ALT) levels in the massive HT group (32.63 ± 13.45 IU/L and 34.50 ± 15.87 IU/L, respectively) compared to the non-HT group (25.65 ± 8.46 IU/L and 27.10 ± 7.83 IU/L), both with *p*-values < 0.001 and 0.002.

**Figure 8:**
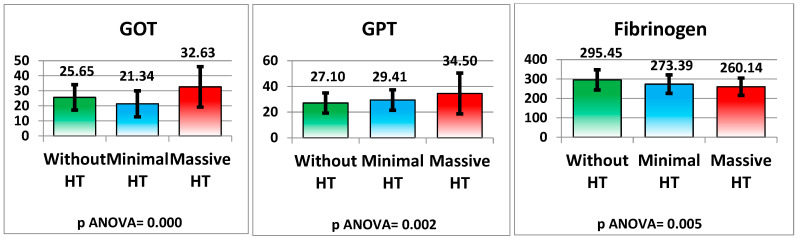
Hepaticchanges.

Fibrinogen levels were significantly lower in both the minimal HT (273.39 ± 47.68 mg/dL) and massive HT (260.14 ± 44.53 mg/dL) groups compared to the non-HT group (295.45 ± 51.79 mg/dL), with a *p*-value of 0.005. This reduction in fibrinogen could reflect a consumptive coagulopathy associated with the HT process (see Figure 8 and Table 2).

**Figure 9:**
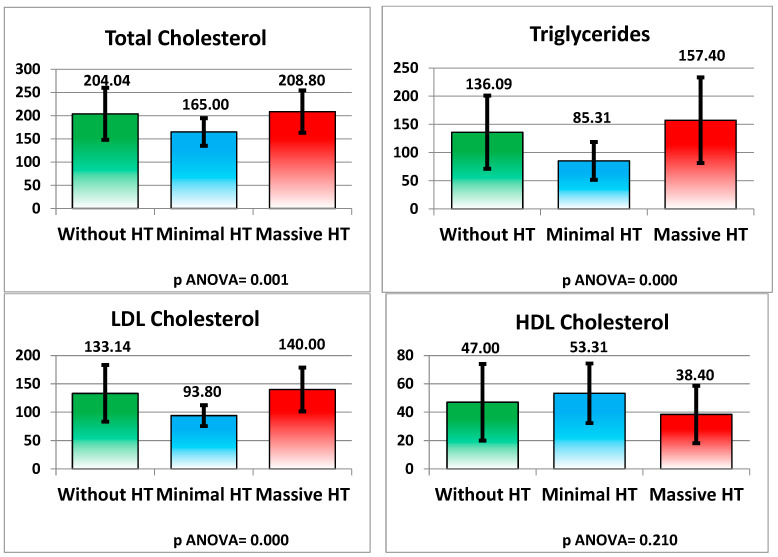
Lipid Profile.

Total cholesterol was lower in the minimal HT group (165.00 ± 29.78 mg/dL) compared to the non-HT (204.04 ± 55.94 mg/dL) and massive HT groups (208.80 ± 45.74 mg/dL), with a *p*-value of 0.001. Triglyceride levels were higher in the massive HT group (157.40 ± 75.88 mg/dL) compared to the minimal HT group (85.31 ± 33.39 mg/dL), with a *p*-value of <0.001. LDL cholesterol was also higher in the massive HT group (140.00 ± 38.72 mg/dL) than in the non-HT (133.14 ± 50.13 mg/dL) and minimal HT groups (93.80 ± 18.44 mg/dL), with a *p*-value of <0.001. No significant difference was found in HDL cholesterol levels (*p* = 0.210) (see Figure 9 and Table 2).

#### 3.6.4. Kidney Function and Blood Pressure

**Figure 10:**
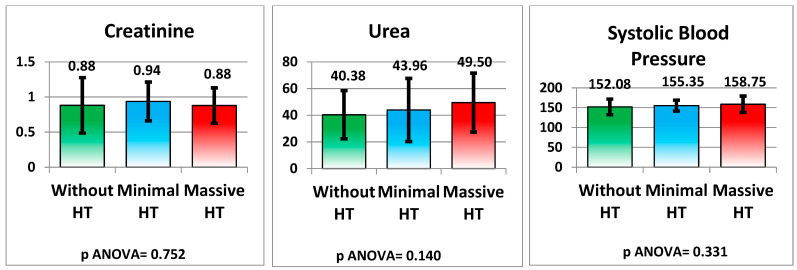
Kidney function and blood pressure.

As measured by creatinine and urea levels, renal function did not show significant differences between the groups (*p* = 0.752 and *p* = 0.140, respectively). Systolic blood pressure was slightly higher in the HT groups but did not reach statistical significance (*p* = 0.331) (see Figure 10 and Table 2).

## 4. Discussion

Hemorrhagic transformation is the most common complication following intravenous thrombolysis with rt-PA [15]. In our study, among the 356 patients analyzed, the overall incidence of HT was 12.08%, with 8.15% classified as minimal HT and 3.93% as massive HT. These findings align with previously reported data in the literature [15,16].

Advancing age is linked to a higher risk of HT [17] and is also associated with poorer stroke outcomes [18]. The increase in the risk of HT with age is multifactorial. Older patients are more likely to have comorbidities such as hypertension, diabetes, and hyperlipidemia, which contribute to systemic inflammation and atherosclerosis. Furthermore, elderly individuals often have a higher burden of cerebrovascular disease and are more likely to be on antithrombotic treatment [8]. This combination of factors, including increased systemic inflammation and compromised blood–brain barrier integrity, enhances the risk of HT following a stroke. In our study, age is a risk factor for hemorrhagic transformation.

High blood pressure can accelerate the damage to brain tissue already affected by infarction and can compromise the blood–brain barrier, thus facilitating intracranial hemorrhage [8,19]. When blood pressure rises significantly during thrombolytic treatment, the risk of post-thrombolysis hemorrhage is much higher, as the fragility of cerebral blood vessels increases, potentially leading to their rupture even in the presence of thrombolytic therapy [20]. In our study, patients with treatment-resistant hypertension were excluded, and our results showed no significant difference between the three groups. This could partly be attributed to the effective therapeutic measures taken to control blood pressure, which may have contributed to reducing the risk of HT in the patients included in the study. When analyzing the diagnosis of arterial hypertension, a higher percentage was observed in patients with HT compared to those without.

Atrial fibrillation is a significant risk factor for ischemic stroke, with approximately 25% of ischemic stroke patients having this condition [21]. Many studies show that the presence of atrial fibrillation predisposes to HT in patients with ischemic stroke [16,22]. In our study, we observed a statistically significant difference, with patients who experienced HT having a higher percentage of atrial fibrillation. Although an elevated INR and the use of new anticoagulants are contraindications for thrombolysis in ischemic stroke, it should not be overlooked that the use of anticoagulants significantly increases the risk of bleeding.

Our study provides insight into the ongoing debate regarding the relationship between obesity and the risk of HT in AIS. The “obesity paradox” has been described in stroke patients, with some studies suggesting that obesity is associated with a more favorable prognosis and a lower risk of HT [23,24,25]. On the other hand, other studies have linked obesity to worse outcomes and increased mortality [26], while some found no significant impact [27,28,29]. In our study, we observed a higher prevalence of obesity among patients with severe HT. Similarly, overweight patients were more frequent in the massive HT group (35.71%) than in the non-HT group (23%). While our results support the notion that obesity may contribute to worse outcomes in AIS, they contrast with the obesity paradox described in some studies.

Although the prolonged QTc interval is commonly observed and is one of the most frequent ECG abnormalities in patients with AIS [30,31], its clinical significance in AIS remains limited due to the complex underlying mechanisms. In addition to neurally mediated autonomic dysregulation, which contributes to QTc interval prolongation [32], other factors common in stroke patients may also play a role [33], including atherosclerotic risk factors, cardiac diseases, electrolyte imbalances, and certain medications. A prolonged QTc interval was linked to a higher risk of long-term mortality, aligning with the increasing prevalence of cardiovascular risk factors and greater stroke severity [30,34,35]. However, no direct studies have been identified linking QTc interval prolongation to the risk of HT in AIS. Our study findings show a statistically significant association between QT interval prolongation and the severity of HT. These results suggest that QT interval prolongation may be indicative of greater stroke severity, potentially contributing to an increased risk of HT.

Hyperglycemia occurs in about 28–40% of patients with AIS [8,36]. It may be linked to pre-existing diabetes mellitus (DM) or a stress-induced response that raises levels of cortisol, glucose, and catecholamines [8]. In both diabetic and non-diabetic patients, hyperglycemia is associated with an increased risk of HT and poorer clinical outcomes [36]. Hyperglycemia can exacerbate inflammation, leading to greater blood–brain barrier (BBB) disruption and an increased risk of HT [37]. Diabetes has been linked to elevated levels of plasma TNF, IL-1b, interleukin 6 (IL-6), interferon-g (IFNg), and PAI-1, as well as changes in immune cell responses, resulting in chronic low-grade inflammation [38,39]. In our study, we observed higher blood glucose levels in patients who experienced HT. This finding further supports the existing literature linking hyperglycemia to an increased risk of hemorrhagic complications following thrombolytic therapy.

In our study, as expected, a lower platelet count predisposes to HT, as demonstrated in other studies [15,16,40]. This is consistent with previous research, which showed that lower platelet levels were associated with early HT in patients treated with intravenous thrombolysis [41,42]. Several studies have suggested that the mean platelet volume and distribution width, both before and after thrombolysis, are associated with hemorrhage following intravenous thrombolysis [43].

As demonstrated in other studies [16], a low fibrinogen level increases the risk of HT, and in our study, those who had HT had a low fibrinogen level. The question remains whether the low fibrinogen level results from consumptive coagulopathy, thrombolysis, or an independent factor predisposing to HT. In the context of ischemic stroke and thrombolysis, fibrinogen levels are important for forming the thrombus and its subsequent response to thrombolytic therapy [44]. As highlighted in previous studies, hyperfibrinogenemia has been linked to an increased risk of venous and arterial thrombosis [45], while reduced fibrinogen levels after stroke are associated with worse neurologic outcomes [46]. Specifically, decreased fibrinogen levels may reflect impaired coagulation, potentially increasing the risk of HT, which is a concern for patients receiving thrombolytic therapy.

Our study adds to the existing evidence on the relationship between lipid levels and HT in AIS. Previous research has linked lower LDL cholesterol levels to symptomatic intracerebral hemorrhage in AIS patients, both with and without reperfusion therapy [47,48,49,50]. Additionally, studies in non-thrombolyzed patients found an association between lower TC and LDL cholesterol levels and an increased risk of HT [51]. A systematic review further supported these findings, showing that patients with HT had lower LDL cholesterol levels, while those with symptomatic intracerebral hemorrhage had lower TC levels [52]. In our study, patients with minimal HT had significantly lower TC and LDL cholesterol levels than those without HT or with massive HT, reinforcing prior findings on the potential role of low lipid levels in HT risk. However, TC and LDL cholesterol levels in massive HT were comparable to or even higher than in patients without HT, suggesting that severe HT may involve additional contributing factors. Triglyceride levels were lowest in the minimal HT group but highest in the massive HT group, possibly indicating a protective or compensatory role. HDL cholesterol, however, showed no significant association with HT. These findings highlight the complex interplay between lipid metabolism and HT, warranting further investigation.

Discussing the relationship between renal dysfunction and HT in the context of ischemic stroke, the literature suggests that patients with uremia are at an increased risk for bleeding complications due to several factors, including uremia-associated bleeding diathesis and comorbid conditions that predispose to bleeding [53]. Additionally, a strong and graded inverse association has been reported between glomerular filtration rate (GFR) and the risk of hemorrhagic stroke, independent of other vascular risk factors [54,55]. In our study, we evaluated renal parameters (creatinine and urea) among patients with and without HT, and the mean values of these markers did not show significant differences between the groups without HT, with minimal HT, and with massive HT (*p* = 0.752 for creatinine and *p* = 0.140 for urea), suggesting that, in our cohort, renal dysfunction did not have a significant impact on the risk of HT. These results may be consistent with some reports that have not found a significant relationship between renal dysfunction and HT after ischemic stroke. However, it is important to note that severe renal dysfunction or extreme uremia may have a different impact depending on the severity of the renal condition and the presence of other comorbidities [56].

As a single-center retrospective study, our findings may be influenced by selection bias and may not be fully generalizable to broader populations. A multicenter prospective study would help validate these results. Additionally, while we aimed to apply more advanced statistical methods, such as logistic regression and survival analysis, we were constrained by the lack of time-to-event data (preventing the use of Cox regression) and the small sample size, which limited the reliability of logistic models. These factors restricted our ability to assess the independent predictive value of certain parameters. Certain clinical parameters, such as smoking status or metabolic factors, may not have been uniformly collected, introducing potential biases in result interpretation. Future studies should ensure more comprehensive data collection. The classification of HT in our study was broad and did not differentiate between hemorrhagic infarction and parenchymal hematoma, which may have limited a more precise correlation with clinical outcomes. Advanced imaging techniques could improve HT characterization in future studies.

## 5. Conclusions

Hemorrhagic transformation is a common complication of AIS and is linked to poor clinical outcomes. Identifying the risk factors for HT may help decrease their occurrence and severity.

The risk factors significantly associated with HT in our study included lower platelet count, elevated blood glucose, prolonged QT interval, reduced fibrinogen levels, and the presence of atrial fibrillation, hypertension, time of symptoms unset, and delayed door-to-needle time. Other contributing factors were diabetes mellitus, dense ACM sign on CT, older age, obesity, early neurological deterioration, QT interval, and elevated GOT and GPT levels, as well as lower LDL cholesterol, total cholesterol, and triglycerides.

Given the multiple factors contributing to HT, early identification of these risk factors, such as metabolic changes, comorbidities, and thrombolysis timing, may help optimize treatment strategies, reduce hemorrhagic complications, and ultimately improve patient outcomes.

## Figures and Tables

**Table 1:** Patient Characteristics by type of hemorrhagic transformation (DTN = door-to-needle time, NHSS = National Institutes of Health Stroke Scale, MCA = middle cerebral artery).

Variable	Without HT (n = 313)	Minimal HT (n = 29)	Massive HT (n = 14)	*p*-Value
Hemorrhagic Transformation	313 (87.92%)	29 (8.15%)	14 (3.93%)	-
Deceased	42 (13.42%)	12 (41.38%)	10 (71.43%)	<0.001
Alive	271 (86.58%)	17 (58.62%)	4 (28.57%)	
Age < 60	56 (17.89%)	0 (0%)	0 (0%)	<0.001
Age 60–69	86 (27.48%)	14 (48.28%)	2 (14.29%)	
Age 70–79	124 (39.62%)	10 (34.48%)	5 (35.71%)	
Age 80–89	47 (15.02%)	5 (17.24%)	7 (50%)	
Hypertension+	168 (53.67%)	22 (75.86%)	12 (85.71%)	0.005
Hypertension−	145 (46.33%)	7 (24.14%)	2 (14.29%)	
Diabetes Mellitus+	67 (21.41%)	8 (27.59%)	8 (57.14%)	0.007
Diabetes Mellitus−	246 (78.59%)	21 (72.41%)	6 (42.86%)	
Atrial Fibrillation+	79 (25.2%)	12 (41.38%)	9 (64.29%)	0.001
Atrial Fibrillation−	234 (74.8%)	17 (58.62%)	5 (35.71%)	
NHSS increased at 2 h/24 h	11 (3.51%)	5 (17.24%)	3 (21.43%)	<0.001
NHSS decreased at 2 h/24 h	302 (96.49%)	24 (82.76%)	11 (78.57%)	
Nutritional Status—Normal	198 (63.26%)	17 (58.62%)	2 (14.29%)	0.001
Nutritional Status—Overweight	84 (23.00%)	7 (24.14%)	5 (35.71%)	
Nutritional Status—Obese	55 (13.74%)	5 (17.24%)	7 (50.00%)	
Smoker+	94 (30.03%)	5 (17.24%)	3 (21.43%)	0.287
Smoker−	219 (69.97%)	24 (82.75%)	11 (78.57%)	
Dense CT MCA+	67 (21.4%)	12 (41.37%)	7 (50%)	0.003
Dense CT MCA−	246 (78.6%)	17 (58.62%)	7 (50%)	
Carotid	289 (92.3%)	29 (100%)	14 (100%)	0.17
Vertebro-basilar	24 (7.7%)	0 (0%)	0 (0%)	
ASPECT < 10	75 (23.9%)	5 (17.24%)	3 (21.43%)	0.70
ASPECT = 10	238 (76.1%)	24 (82.76%)	11 (78.57%)	
Symptom Onset < 2 h	131 (41.99%)	3 (10.34%)	2 (14.29%)	<0.001
Symptom Onset 2–3 h	87 (27.88%)	4 (13.79%)	3 (21.43%)	
Symptom Onset 3–4 h	66 (21.15%)	14 (48.28%)	4 (28.57%)	
Symptom Onset 4–5 h	29 (9.29%)	8 (27.59%)	5 (35.71%)	
DTN < 60 min	186 (59.42%)	8 (27.59%)	5 (35.71%)	<0.001
DTN > 60 min	127 (40.58%)	21 (72.41%)	9 (64.29%)	

**Table 2:** Laboratory and clinical parameters by type of hemorrhagic transformation.

Variable	Without HT (Mean ± SD)	Minimal HT (Mean ± SD)	Massive HT (Mean ± SD)	*p*-Value
QT Interval (ms)	449.87 ± 47.04	467.47 ± 25.85	483.75 ± 21.24	0.004
Blood Glucose (mg/dL)	139.58 ± 54.46	170.88 ± 58.43	166.75 ± 64.43	0.004
Platelets (cells/μL)	218,575.42 ± 58,885.04	194,235.29 ± 57,919.70	172,625.00 ± 50,793.52	0.003
INR (International Normalized Ratio)	1.12 ± 0.18	1.11 ± 0.10	1.20 ± 0.10	0.186
GOT (AST) (U/L)	25.65 ± 8.46	21.34 ± 8.66	32.63 ± 13.45	<0.001
GPT (ALT) (U/L)	27.10 ± 7.83	29.41 ± 7.95	34.50 ± 15.87	0.002
Fibrinogen (mg/dL)	295.45 ± 51.79	273.39 ± 47.68	260.14 ± 44.53	0.005
Total Cholesterol (mg/dL)	204.04 ± 55.94	165.00 ± 29.78	208.80 ± 45.74	0.001
Triglycerides (mg/dL)	136.09 ± 64.97	85.31 ± 33.39	157.40 ± 75.88	<0.001
LDL Cholesterol (mg/dL)	133.14 ± 50.13	93.80 ± 18.44	140.00 ± 38.72	<0.001
HDL Cholesterol (mg/dL)	47.00 ± 27.00	53.31 ± 21.06	38.40 ± 20.23	0.210
Creatinine (mg/dL)	0.88 ± 0.40	0.94 ± 0.28	0.88 ± 0.25	0.752
Urea (mg/dL)	40.38 ± 18.05	43.96 ± 23.69	49.50 ± 22.12	0.140
Systolic Blood Pressure (mmHg)	152.08 ± 19.78	155.35 ± 13.74	158.75 ± 20.72	0.331

## Data Availability

The datasets generated during and/or analyzed during the current study are available from the corresponding author upon reasonable request.
